# Toxoplasma gondii LCAT Primarily Contributes to Tachyzoite Egress

**DOI:** 10.1128/mSphereDirect.00073-18

**Published:** 2018-02-28

**Authors:** Aric J. Schultz, Vern B. Carruthers

**Affiliations:** a Department of Microbiology and Immunology, University of Michigan School of Medicine, Ann Arbor, Michigan, USA; University at Buffalo; Boston College; Indiana University School of Medicine

**Keywords:** Toxoplasma gondii, egress, gene disruption, phospholipase

## Abstract

Egress is a crucial phase of the Toxoplasma gondii intracellular lytic cycle. This is a process that drives inflammation and is strongly associated with the pathogenesis observed during toxoplasmosis. Despite the link between this process and virulence, little is known about egress on a mechanistic or descriptive level. Previously published work has suggested that a phospholipase, lecithin-cholesterol acyltransferase (LCAT), secreted from the parasite’s dense granules contributes to parasite growth, virulence, and egress. Here we present evidence from several independent mutant parasite lines confirming a role for LCAT in efficient egress, although no defects in growth or virulence were apparent. We also show via genetic complementation that the catalytic activity of LCAT is required for its role in parasite egress. This work solidifies the contribution of LCAT to egress of T. gondii tachyzoites.

**IMPORTANCE** Toxoplasma gondii is one of the most successful human pathogens, infecting an estimated 2.5 billion people across the globe. Pathogenesis seen during acute or reactivated toxoplasmosis has been closely tied to the parasite’s intracellular lytic life cycle, which culminates in an event called egress that results in the release of freshly replicated parasites from the infected host cell. Despite the highly destructive, cytolytic nature of this event and its downstream consequences, very little is known about how the parasite accomplishes this step. Previous work has suggested a role for a secreted phospholipase, LCAT, in *Toxoplasma* egress and roles in cell traversal and egress in the *Plasmodium* species orthologue. We confirm here that LCAT-deficient tachyzoites are unable to efficiently egress from infected monolayers, and we provide evidence that LCAT catalytic activity is required for its role in egress.

## Abstract (precis)

Toxoplasma gondii is one of the most successful human pathogens, infecting an estimated 2.5 billion people across the globe. Pathogenesis seen during acute or reactivated toxoplasmosis has been closely tied to the parasite’s intracellular lytic life cycle, which culminates in an event called egress that results in the release of freshly replicated parasites from the infected host cell. Despite the highly destructive, cytolytic nature of this event and its downstream consequences, very little is known about how the parasite accomplishes this step. Previous work has suggested a role for a secreted phospholipase, LCAT, in *Toxoplasma* egress and roles in cell traversal and egress in the *Plasmodium* species orthologue. We confirm here that LCAT-deficient tachyzoites are unable to efficiently egress from infected monolayers, and we provide evidence that LCAT catalytic activity is required for its role in egress.

## INTRODUCTION

Toxoplasma gondii is an obligate intracellular parasite that infects a wide range of host animals, including humans. This parasite is estimated to infect up to one in three people worldwide (1), who are presumed to harbor the infection for the remainder of their lifetime. *Toxoplasma* commonly infects humans congenitally, through the ingestion of oocysts shed in the feces of infected felines, or from consumption of undercooked meat that contains tissue cysts (2). For T. gondii and other intracellular pathogens, egress represents a crucial step of the life cycle, which must occur in order for the parasite to infect new host cells. With this in mind, it is not surprising that intracellular microbes have evolved numerous approaches and strategies to complete this task (3). These different approaches have vastly different impacts on the host and the damage response to infection (4). One such strategy utilized by protozoan parasites and extensively within *Toxoplasma*’s apicomplexan phylum is cytolytic egress. This event in particular results in massive release of inflammatory signals from cell death and tissue destruction. The resulting pyrogenic immune response is a hallmark of both toxoplasmosis and malaria, caused by the related apicomplexan Plasmodium falciparum. While infection of a healthy adult is typically benign, toxoplasmosis can be disastrous among immunocompromised patients or those infected congenitally. Common manifestations of pathogenesis seen include potentially fatal toxoplasmic encephalitis and retinal destruction, frequently observed among congenitally infected individuals (5–7). Indeed, the importance of controlling the host immune response is illustrated while examining the devastating effect of toxoplasmosis on the mortality of HIV-AIDS patients in the preantiretroviral (highly active antiretroviral therapy [HAART]) era (8–10), an observation that underscores the need for robust monitoring of tissue transplant and/or chemotherapy patients today.

At the center of the pathogenesis seen during toxoplasmosis is the lytic cycle of *Toxoplasma*. This cycle begins with the active invasion of the parasite into a new host cell and the formation of an intracellular replicative niche called the parasitophorous vacuole (PV) formed from the host plasma membrane during invasion (11). After replication within the sequestered PV, the tachyzoites actively egress from the host cell and begin the cycle anew by invading nearby host cells. In many aspects, invasion and egress are similar events and rely on secretion of the parasite’s micronemes and parasite motility, both processes dependent on activation of intracellular calcium signaling pathways (12). Egress requires the breakdown of two notable membrane barriers: the parasitophorous vacuolar membrane (PVM) and the host plasma membrane (HPM). However, outside the microneme-derived perforin-like protein 1 (PLP1), which permeabilizes the PVM (13), effector molecules directly involved in this process have not been thoroughly defined.

Lecithin-cholesterol acyltransferase (LCAT) was originally described in Plasmodium berghei as a phospholipase named PL in a screen to identify sporozoite proteins involved in the establishment of the infection (14). More recently, this phospholipase has been tied to permeabilization of the PVM during *Plasmodium* merozoite egress from hepatocytes (15). Since then, additional work has been published focusing on the *Toxoplasma* orthologue LCAT. A mutant strain lacking LCAT showed slower growth based on fewer parasites per PV and smaller plaque area, a defect in egress, and a notable loss in virulence manifested by 70% survival of mice infected with mutant strains versus 0% survival of those infected with the parental strain (16).

To understand in greater detail the secreted effectors that Toxoplasma gondii utilizes during egress, we have focused our attention on T. gondii LCAT (TgLCAT), hereafter referred to simply as “LCAT.” During the course of this study, we consistently observed that parasites lacking LCAT were unable to complete the egress event as efficiently as their wild-type counterparts. However, unlike the previously described LCAT mutant (16), the new strains show normal parasite replication *in vitro* and no loss of virulence in mice. These findings independently validate a role for LCAT during parasite egress.

## RESULTS

### Genetic removal and complementation of LCAT.

**FIG 1:**
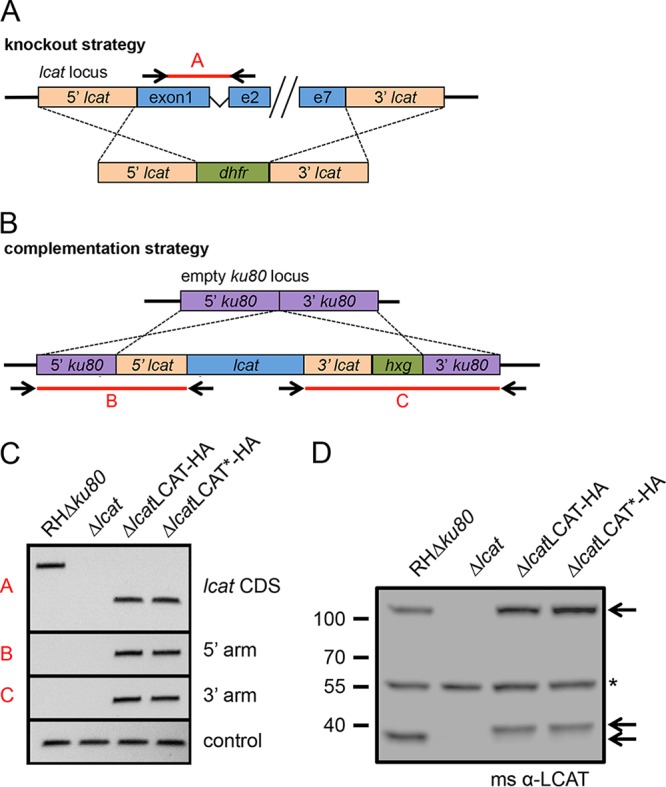
Generation of Δ*lcat* and Δ*lcat* complemented lines. (A) Schematic representation of the linear knockout construct and generation of the LCAT knockout by double homologous integration of *dhfr* at the *lcat* locus. e2, exon 2. (B) Schematic representation of the complementation of the Δ*lcat* strain, showing the *lcat* cDNA under direction of its endogenous 5′ and 3′ untranslated regions (UTRs), being driven to integrate into the “empty” *ku80* locus by double homologous recombination. (C) PCR showing genetic confirmation of the knockout and complemented strains by amplification of the coding sequence (CDS) (primer pair A), and detection of the 5′ and 3′ arms of the complementation construct within the *ku80* locus (primer pairs B and C, respectively). (D) Western blot confirming the loss of and restoration of LCAT expression in the knockout and complemented strains, respectively. The positions of molecular mass markers (in kDa) are indicated to the left of the blot. The arrows to the right of the blot indicate specific full-length and proteolytically processed bands (slightly shifted in the complemented strains, due to the addition of HA epitope tag). The asterisk denotes a nonspecific band. α-LCAT, anti-LCAT. ms, mouse (indicating the species of antibody used).

Previous work regarding LCAT focused on parasites that were genetically ablated in the wild-type RH background. To assess the function of LCAT independently, we remade the LCAT knockout in the RHΔ*ku80* line, which allows more precise gene deletion and complementation (17). The LCAT locus (EuPathDB TGME49_272420) was modified by double homologous integration to replace the LCAT-coding region with the dihydrofolate reductase (*dhfr*) selectable marker. After transfection of a linear knockout construct (Fig. 1A) constructed by fusion PCR and pyrimethamine selection, the absence of LCAT was confirmed genetically via PCR amplification of the coding sequence and at the protein level as seen by Western blotting (Fig. 1C and D). This RHΔ*ku80Δlcat* strain was then complemented with C-terminally hemagglutinin (HA)-tagged wild type (WT) gene (Δ*lcat*LCAT-HA) and a mutant allele (Δ*lcat*LCAT*-HA), which harbors an S332A point mutation predicted to destroy catalytic activity from the active site serine. These constructs were integrated into the “empty” *Δku80* locus by double homologous recombination (Fig. 1B), and incorporation into the genome was confirmed by PCR detection of the shortened cDNA within the *ku80* locus and by restoration of protein expression, albeit slightly shifted in size due to the addition of the epitope tag (Fig. 1C and D).

### LCAT mutants show normal plaque formation and replication.

**FIG 2:**
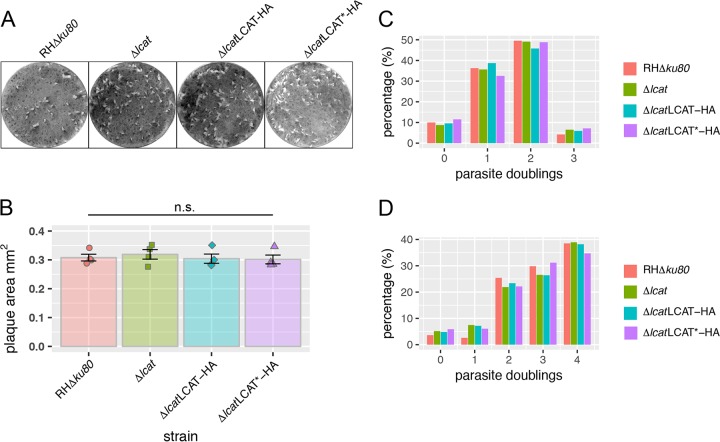
LCAT does not play a role in growth or replication. (A) LCAT knockout and complemented strains form normal plaques over a 7-day infection of HFFs. (B) Quantification of the plaque area via ImageJ showed no significant differences between parental, knockout, or complemented strains. Data shown are means ± standard errors of the means (SEM) (error bars) from three or four biological replicates. The values were not significantly different (n.s.). (C and D) Quantification of parasite replication by counting parasites per vacuole at 16 h (C) or 32 h (D). Data represent pooled data from three biological replicates, each with at least 250 vacuoles per strain counted. No significant differences were found using Pearson’s chi-squared test.

We next performed plaque assays to test the LCAT-deficient parasites for any broad defects through successive lytic cycles. Confluent monolayers of human foreskin fibroblasts (HFFs) were infected, and the parasites were allowed to replicate undisturbed for 7 days before fixation and staining of the monolayer (Fig. 2A). Quantification of the resulting plaque sizes showed no difference between parental, knockout, or either one of the complemented strains (Fig. 2B). These results indicate the absence of LCAT does not affect the ability of tachyzoites to efficiently progress through the lytic cycle *in vitro*. While the results were not suggestive of a growth defect, we shifted to an intracellular replication assay to examine the rate of parasite division between strains more precisely. Parasite replication was directly measured by infecting confluent monolayers of HFFs and allowing replication to occur for either 16 or 32 h before fixation and manual quantification of parasites per vacuole via fluorescence microscopy. No differences in the replication rate of Δ*lcat* parasites compared to parental or complemented strains were found (Fig. 2C and D).

### LCAT-deficient parasites display normal virulence in the murine model.

**FIG 3:**
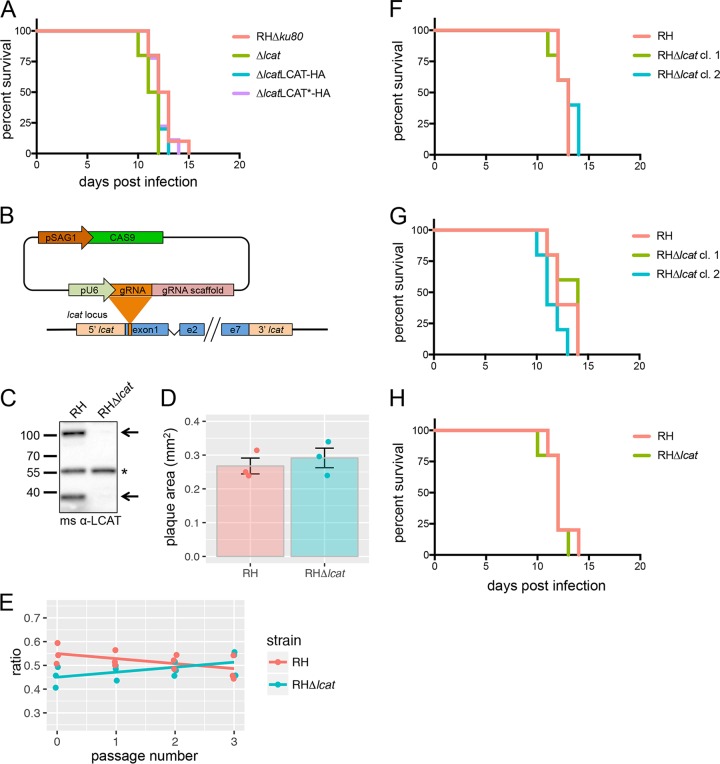
LCAT does not contribute to virulence in the murine model. (A) Survival data of infected mice. Female BALB/c mice were infected subcutaneously with 10 tachyzoites of the indicated strain, and survival was monitored over the following weeks. The data represent 10 mice per group pooled from two biological replicates. (B) Schematic illustrating the use of CRISPR-Cas9 for targeted disruption of the *lcat* coding sequence. A 20-bp sgRNA was used to direct the nuclease to the 5′ region of exon 1. (C) Western blot confirming the loss of LCAT expression after CRISPR-Cas9-mediated disruption. The asterisk denotes a nonspecific band. (D) Quantification of plaque area in wild-type RH and RHΔ*lcat*. (E) Parasite growth as measured by an *in vitro* competition assay showing the relative abundance of RH and RHΔ*lcat* across three serial passages. (F) Survival of female BALB/c mice infected with 10 tachyzoites subcutaneously. (G) Survival of female BALB/c mice infected with 50 tachyzoites subcutaneously. Data in panels F and G are from 10 mice per group pooled from two biological replicates infected with wild-type or two separate clones of RHΔ*lcat*. (H) Survival of female outbred Swiss Webster mice infected with 10 tachyzoites subcutaneously. The data are from five mice in one experiment. No significant differences were observed in growth (Student’s *t* test) or survival (Kaplan-Meier analysis).

We next sought to measure virulence of the new Δ*lcat* strain in the acute model of murine infection. Due to the highly virulent nature of the type I strain background, we chose to use a low dose of 10 tachyzoites administered subcutaneously to BALB/c mice. By 10 days postinfection, infected mice were displaying signs of morbidity, and the mice were euthanized by 10 to 15 days postinfection. No statistically significant differences in survival were seen, as mice infected with parental, knockout, or complemented strains succumbed to the infection with similar kinetics. To investigate a potential role for LCAT *in vivo* more thoroughly, we recreated the knockout in the wild-type RH background. LCAT-deficient parasites were generated using the clustered regularly interspaced short palindromic repeat (CRISPR)-Cas9 system (18) by targeting the Cas9 nuclease via a single guide RNA (sgRNA) to a region near the 5′ end of *lcat* exon 1 (Fig. 3B). After transfection of the plasmid containing the *lcat* sgRNA, we were able to isolate parasite clones that no longer expressed the LCAT protein, as confirmed by Western blotting shown for one such clone (Fig. 3C). No difference in plaque area was observed between wild-type and RHΔ*lcat* tachyzoites, confirming that LCAT-deficient parasites progress normally through the lytic cycles, similar to our initial observations in the Δ*ku80* background (Fig. 3D). In lieu of further quantification by counting parasites per vacuole as done previously, we chose to use an *in vitro* competition assay which, over serial passages, should be a more sensitive method of quantifying parasite growth. Briefly, RHΔ*lcat* parasites were transfected with a plasmid allowing for stable expression of green fluorescent protein (GFP) to allow for easy identification and differentiation of RH versus RHΔ*lcat* parasites. Both parental and knockout parasites were inoculated into T25 flasks of confluent HFFs, and after each lysis, parasites were collected for quantification via fluorescence microscopy. The results of the *in vitro* competition assay mirror what was seen in previous growth assays, as there were no significant differences in relative abundance after serial coculture (Fig. 3E).

To measure virulence of the new RHΔ*lcat* strain, we injected BALB/c mice subcutaneously with 10 or 50 tachyzoites of wild-type RH or two clones of RHΔ*lcat*. Again, no statistically significant differences were elucidated between any of the strains (Fig. 3F and G). This experiment was repeated in outbred mice, as we infected Swiss Webster mice with 50 tachyzoites of wild-type RH or a single clone of RHΔ*lcat* with very similar results (Fig. 3H). Collectively, after testing the LCAT knockout from multiple parasite strain backgrounds in the context of multiple mouse strain backgrounds, our findings suggest that LCAT does not contribute to *Toxoplasma* virulence.

### Parasites lacking LCAT display a consistent phenotype in impaired egress.

**FIG 4:**
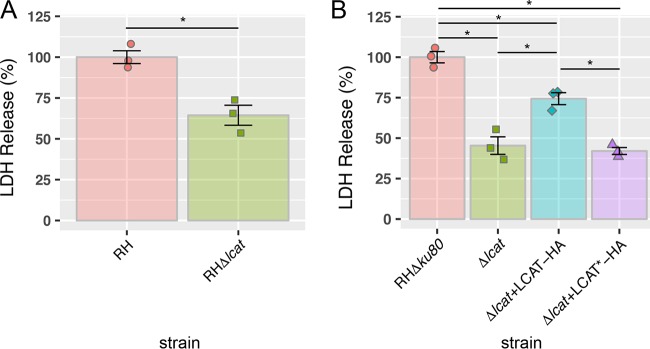
LCAT-deficient parasites are unable to efficiently egress from host cells. (A and B) Egress following 20 min of incubation with 57 µM zaprinast. Lactate dehydrogenase release following induction was used as a measure for egress and normalized to wild-type RH (A) or RHΔ*ku80* (B). Statistical significance was assigned by Student’s *t* test. Data shown are means ± SEM (error bars) from three biological replicates each with three technical replicates. Values that are significantly different (*P* < 0.05) by Student’s *t* test are indicated by a bar and asterisk.

We next examined to what extent the loss of LCAT expression affects parasite egress from the host cell. To determine this, we infected HFF monolayers in 96-well plates with either WT (RH or RHΔ*ku80*) or knockout (RHΔ*lcat* or RHΔ*ku80*Δ*lcat*) parasites. After 30 h of replication, the infected monolayers were then treated with zaprinast to induce egress pharmacologically. Zaprinast is a phosphodiesterase inhibitor that induces egress by activation of the parasite protein kinase G (PKG) (19). The culture supernatant was collected and assayed for lactate dehydrogenase (LDH) content as a function of egress (host membrane damage). The results showed ~40% and ~55% in zaprinast-induced LDH release by RHΔ*lcat* and RHΔ*lcatΔku80* parasites, respectively (Fig. 4A and B). This phenotype was partially, yet significantly, restored upon complementation of the knockout with the wild-type version of LCAT but not with the catalytically inert mutant (Fig. 4B). These findings confirm a role for LCAT in *Toxoplasma* egress and newly identify a requirement for LCAT enzymatic activity in this event.

## DISCUSSION

As obligate intracellular pathogens, egress from the infected host cell is an absolute requirement for apicomplexan parasites. Therefore, it comes as no surprise that these parasites have evolved a multitude of specialized effector molecules to complete this task. While currently only one such protein has been mechanistically described in *Toxoplasma* (PLP1) (13, 20, 21), there have been suggested roles for a genetically duplicated secreted nucleoside triphosphate hydrolase (22) that could act in signaling or as an effector. Host calpain proteases have also been implicated, which are thought to facilitate egress by selective degradation of the host cytoskeleton prior to egress (23, 24). The landscape of secreted egress effectors has become somewhat better defined in the *Plasmodium* field, with roles being ascribed for secreted proteases such as SUB1, members of the SERA family, and DPAP3 (25–27) in addition to several members of the perforin-like protein family (28–30).

In 2005, a surface-localized phospholipase, Plasmodium berghei phospholipase (PbPL), was described in P. berghei, which facilitates sporozoite cell traversal (14), and was more recently shown to aid in merozoite egress from infected hepatocytes (15). Subsequently, the *Toxoplasma* orthologue LCAT was shown to have a similar role in egress of tachyzoites *in vitro* (16). In a continuation of that study, we have found a consistent role for LCAT in parasite egress but were not able to identify a supporting role in parasite replication or a noticeable role *in vivo*. One possible explanation is that the original LCAT knockout was made in the RH strain, which can exhibit off-target integration of knockout constructs. Replication and virulence phenotypes in the original LCAT knockout were reversed; however, complementation was achieved by integration of multiple copies of the expression construct, resulting in marked overexpression of LCAT. We assessed replication and virulence in two independent knockouts, including one created in the RHΔ*ku80* background, which allows more precise disruption and complementation. We cannot rule out the possibility that replication and virulence phenotypes in the new Δ*lcat* strains were blunted by compensatory changes in expression of other genes that dictate egress. However, a recent genome-wide mutagenesis screen revealed that parasites lacking LCAT were not at a competitive disadvantage in culture (31), supporting the conclusion that LCAT does not contribute substantially to parasite replication.

Work on *Plasmodium* describing PbPL has shown an interesting observation of differential localization: sporozoites express PL on the surface, while in developing merozoites, PL localizes to the PVM (14, 15). This differential localization is consistent with the role of PbPL in sporozoite disruption of the HPM during cell traversal and merozoite rupture of the PVM during egress from hepatocytes. Work from the Coppens lab has shown a similarly interesting dual localization: soluble within the PV during intracellular replication and shifting to the parasite surface following egress (16). We attempted to visualize this apparent relocalization of LCAT but were unable to do so due to the following. (i) Anti-HA antibodies failed to detect HA-tagged LCAT (LCAT-HA) by immunofluorescence staining. (ii) Our antibodies made against recombinant LCAT also did not work for immunofluorescence. (iii) LCAT endogenously tagged with a C-terminal yellow fluorescent protein (YFP) fusion showed an egress defect, suggesting that placement of a large tag at this location compromised LCAT function. Nevertheless, if LCAT redistribution occurs, several different scenarios can be envisioned for how LCAT contributes to egress.

In one scenario, LCAT phospholipase activity principally functions by aiding parasite disruption of host-derived membranes, namely, the PVM and HPM. Disrupting such membranes could also indirectly facilitate egress of the parasite by (i) releasing unknown egress factors from sequestration in the parasitophorous vacuole or (ii) allowing the influx of host/environmental factors that stimulate egress. For example, disruption of the HPM and PVM would allow influx of serum components, including serum albumin, which enhances parasite calcium signaling, microneme secretion, and gliding motility via activation of PKG (32).

In another scenario, LCAT could function on the parasite surface by generating products that enhance microneme secretion. LCAT phospholipase A2 activity generates lysophospholipids and fatty acids such as arachidonic acid (AA). In mammalian cells, AA activates plasma membrane AA-regulated Ca^2+^ (ARC) entry channels, encoded by Orai2 and Orai3 proteins (33). Downstream metabolites of AA also activate certain plasma membrane transient potential channels, including TRPV4 (34). Additional studies are needed to distinguish the site of LCAT action and its precise role in egress. However, based on our data, it seems that catalytic activity of LCAT is necessary for its contribution to egress (Fig. 4B), as complementation of the knockout with a catalytically dead mutant does not restore the phenotype. This observation makes it unlikely that LCAT is functioning in a scaffolding or structural role for additional proteins during egress.

Another interesting aspect of this study is that an egress effector secreted constitutively from the dense granules must somehow be functionally regulated, so as not to induce premature egress of the tachyzoites. Two potential methods of regulation are proteolytic processing and pH. Recently, a subset of dense granule proteins were shown to be processed at PEXEL motifs by the Golgi resident aspartyl protease ASP5 (35). LCAT contains a putative PEXEL motif, RRLEE starting at amino acid 594, and cleavage at this site would create proteolytic products of approximately the size seen herein (Fig. 1D). Additionally, cleavage of PEXEL-containing motifs has been purported to drive localization to the PVM (36). As for regulation by pH, LCAT is related to lysosomal phospholipase A2 (LPLA2), and it possesses a canonical LPLA2 lipase motif, AXSXG. LPLA2 enzymes are activated by low pH, which promotes binding to target membranes (37). This coupled with a recent report suggesting acidification of the PV near the time of egress (20) raises the possibility that LCAT activity is stimulated by low pH in a manner similar to LPLA2.

Finally, it remains to be seen whether a cooperative function exists between LCAT and other proteins that influence egress, such as PLP1 and GRA41. As described by Hybiske and Stephens (3), many intracellular pathogens coopt pore-forming toxins (PFTs) and phospholipases to escape membrane entrapment. Also worth noting are the parallels we see in *Plasmodium* spp., with stage-specific expression of different PFTs and stage-specific activity of PbPL (sporozoite transmigration versus merozoite egress). While in these systems a direct role has not been established for cooperation between these two molecules, there are established examples of PFTs directly enhancing phospholipase activity, as is the case in bee venom (38). As for GRA41, a recent study showed that a mutation in this dense granule protein results in aberrations of parasite calcium levels and egress. Parasites lacking GRA41 exhibit dysregulation of calcium ion uptake, leading to altered calcium ion homeostasis and premature egress (39). Although LCAT and GRA41 appear to affect egress in opposite ways, they both reside in the PV, suggesting an emerging role for PV resident proteins in *Toxoplasma* egress.

## MATERIALS AND METHODS

### Ethics statement.

This study was carried out in strict accordance with the Public Health Service Policy on Humane Care and Use of Laboratory Animals and Association for the Assessment and Accreditation of Laboratory Animal Care guidelines. The animal protocol was approved by the University of Michigan’s Committee on the Use and Care of Animals (Animal Welfare Assurance A3114-01, protocol 09482). All efforts were made to minimize pain and suffering of the mice.

### Parasite culture.

*Toxoplasma* tachyzoites were maintained by serial passaging and growth in human foreskin fibroblast (HFF) cells. Cell cultures were grown in Dulbecco’s modified Eagle medium (DMEM, Gibco) supplemented with 10% fetal bovine serum (FBS, Gibco), 2 mM glutamine, and 10 mM HEPES and grown in 5% CO_2_ at 37°C. Parasites were liberated by scraping the infected HFF monolayer and passage through a 27-gauge needle. The liberated parasites were then filtered through a 3-µm size filter (Millipore), counted on a hemocytometer, and added to HFFs at the appropriate density. For routine culture of the parasites, 5 drops of naturally egressed parasites were passed into fresh host cells in a T25 flask.

### Creation of transgenic strains.

The knockout construct was constructed via fusion PCR. 5′ and 3′ homologous flanks were amplified from RH genomic DNA (gDNA) using primer pair ajsP1 plus ajsP22 and primer pair ajsP6 plus ajsP23, respectively. The *dhfr* selectable marker was amplified from pYFP.LIC.DHFR (Addgene) with primer pair ajsP20 plus ajsP21. These three products were used as the template and fused via a final PCR to primers ajsP2 and ajsP5. The linear vector was transfected into RHΔ*ku80* parasites, and stable clones were isolated based on pyrimethamine resistance. The complement construct (pLCAT.Ku80.HXG) was created via Gibson cloning (In-Fusion; Clontech). The vector pM2AP.Ku80.HXG was prepared by double digestion with AscI plus SpeI and purified by gel extraction (Qiagen). The 5′ and 3′ *lcat* flanking sequences were amplified from RH gDNA with primer pair ajsP62 plus ajsP52 and primer pair ajsP63 plus ajsP59, respectively. The *lcat* cDNA was amplified from a cDNA library with primer pair ajsP53 plus ajsP58. The catalytically dead construct, pLCATs332a.Ku80.HXG, was generated using site-directed mutagenesis (QuikChange XL; Agilent Technologies) and primers ajsP64 plus ajsP65. The constructs were linearized prior to transfection by double digestion with KpnI plus ApaLI and transfected into RHΔ*ku80Δlcat* parasites, and stable clones were isolated based on resistance to mycophenolic acid (MPA) plus xanthine (Xan). For creation of RHΔ*lcat* lines using CRISPR-Cas9, 20 bp of *lcat*-specific guide sequence was inserted into pCRISPR-Cas9-Ble, using site-directed mutagenesis and the primer pair ajsP200 plus ajsP201. PCR was used for genetic confirmation shown in Fig. 1 as follows: amplification across exon 1 and exon 2 (indicated by the red A in Fig. 1A) with primers ajsP15 plus ajsP138, integration of the 5′ end of the complement construct at the *ku80* locus (indicated by the red B in Fig. 1B) with primers XhoI_Ku80_5\’Flank.f plus ajsP138, and integration of the 3′ end of the complement construct at the *ku80* locus (indicated by the red C in Fig. 1B) with primers ajsP19 plus XhoI_Ku80_5\’Flank.r.

### Primers.

The following primers were used in this study: ajsP1, CTGCATGGGACACAAACAGT; ajsP2, TCGTCACAGCCATCGAAATC; ajsP5, TCGGTCACTGAGCAGCTAG; ajsP6, CAGTTACTTTCAGATCCAACC; ajsP15, ATGGACTTCCTCTCTGGAGG; ajsP19, AGGCTCGAGGAGGATGTTTA; ajsP20, GAGGTCGACGGTATCGATAA; ajsP21, TAGAACTAGTGGATCCCCCT; ajsP22, TTATCGATACCGTCGACCTCTCTGGAAGGAGCGGAACAC; ajsP23, AGGGGGATCCACTAGTTCTATAGAGGGAACTGAGTCGGGA; ajsP52, CCTCCAGAGAGGAAGTCCATTTCTGGAAGGAGCGGAACACT; ajsP53, AGTGTTCCGCTCCTTCCAGAAATGGACTTCCTCTCTGGAGG; ajsP58, CGTAGTCCGGGACGTCGTACGGGTACGTGCTGTCTGCCATAATCG; ajsP59, GTACGACGTCCCGGACTACGCGTAATAGGAGGGAACTGAGTCGGGA; ajsP62, CTTGGTCGTAAGAGAAGAGGAGCGCCCTGGAACAACATAACACA; ajsP63, ATTCGCACCCTCCAAACTAGTGGTCCGAGCGTTTGTTGCAA; ajsP64, AAAGTTGACCTGATCGCCCACGCCTTGGGCAGCATTATTCTGTGT; ajsP65, ACACAGAATAATGCTGCCCAAGGCGTGGGCGATCAGGTCAACTTT; ajsP138, CGTAGGTGGCGTTCATGTAGTAGTCTAGGT; ajsP142, AGGCGACGACAAAGCCGGATCCTCGAGCATAT; ajsP143, GAGGATCCGGCTTTGTCGTCGCCTTTCTCC; ajsP144, GACTACGCGTAAGCTAACAAAGCCCGAAAG; ajsP145, TTTGTTAGCTTACGCGTAGTCCGGGACGTC; ajsP200, GGCACTGGCAAACCAAAGAAGTTTTAGAGCTAGAAATAGC; ajsP201, TTCTTTGGTTTGCCAGTGCCAACTTGACATCCCCATTTACC; XhoI_ku80_5\’Flank.f, GACTCGAGTGGAGATCCAAGCGAGGACTTA; and XhoI_ku80_5\’Flank.r, GACTCGAGGCTTCGAGTCGTCTGTTCTGG.

### Plaque assay.

Infected monolayers in a T25 flask were washed three times with 37°C phosphate-buffered saline (PBS) prior to scraping and host lysis as described above. Parasite suspensions were made via serial 10-fold dilutions to reach a concentration between 75 and 150 parasites/150 µl PBS. Monolayers of HFFs in individual six-well plates were immediately inoculated with 150 µl of the parasite solution. The infected monolayers were then incubated, undisturbed, for 7 days prior to fixation with 2% crystal violet. Plaques were quantified using ImageJ on wells that had been digitally scanned with a ruler included for scale.

### Replication assay.

Infected HFFs were fixed with 4% paraformaldehyde at either 16 or 32 h postinfection. The fixed monolayers were permeabilized with 0.01% Triton X-100 and labeled with rabbit anti-SAG1 and counterstained with 4′,6′-diamidino-2-phenylindole (DAPI). At least 250 vacuoles were quantified per strain per experiment.

### Generation of LCAT-specific polyclonal antibodies.

The bacterial expression construct pET15b was linearized with inverse PCR (iPCR) with primer pair ajsP142 plus ajsP144. The *lcat* coding sequence was amplified with primers ajsP143 plus ajsP145 from a *Toxoplasma* cDNA library and was subcloned into pET15b via Gibson assembly.

### Virulence assay.

Groups of five female BALB/c or Swiss Webster (Jackson) female mice, aged 6 weeks, were infected with either 10 or 50 T. gondii tachyzoites subcutaneously in 150 µl of PBS. Delivery of an accurate dose of infectious parasites was confirmed by performing plaque assays in parallel (described above). In the event of mice surviving the infection, seropositivity was tested by enzyme-linked immunosorbent assay (ELISA).

### *In vitro* competition assay.

RHΔ*lcat* parasites were transfected with a glycosylphosphatidylinositol (GPI)-anchored green fluorescent protein (GFP) variant, and stable clones were selected following treatment with pyrimethamine. Extracellular parasites were counted on a hemocytometer, and roughly equal numbers (5 × 10^5^) of both RH and RHΔ*lcat*GFP parasites were coinoculated into a T25 flask of HFFs. Following lysis of the host cell monolayer, extracellular parasites were collected, filtered, and fixed on poly-l-lysine-coated glass slides. Parasites were immunolabeled with rabbit anti-SAG1 and enumerated via fluorescence microscopy. This process was repeated for a total of three passages.

### Egress assay (LDH).

Thirty hours prior to assay, infected monolayers in a T25 flask were washed three times with 37°C PBS prior to scraping and host lysis as described above. Following centrifugation, parasites were resuspended to a density of 5 × 10^5^/ml, and 100 µl was used per well (5 × 10^4^ tachyzoites). Prior to egress assays, infected wells were washed three times with 37°C Ringer’s buffer. After the final wash, the cells were treated with either 57 µM zaprinast diluted in Ringer’s buffer or Ringer’s solution with an equal volume of dimethyl sulfoxide (DMSO). The treated plates were allowed to incubate at 5% CO_2_ and 37°C for 20 min before removal and immediate placement on ice. Fifty microliters of the supernatant was removed and placed into individual wells of a 96-well round-bottom plate. The round-bottom plates were centrifuged at 4°C at 500 × *g* for 5 min. Thirty microliters of solution was removed, stored at 4°C, and used within 1 h as source assay material for lactate dehydrogenase (LDH) content (BioVision).
